# The Incidence Characteristics of Second Primary Malignancy after Diagnosis of Primary Colon and Rectal Cancer: A Population Based Study

**DOI:** 10.1371/journal.pone.0143067

**Published:** 2015-11-16

**Authors:** Xu Guan, Yinghu Jin, Yinggang Chen, Zheng Jiang, Zheng Liu, Zhixun Zhao, Peng Yan, Guiyu Wang, Xishan Wang

**Affiliations:** 1 Department of Colorectal Surgery, the Second Affiliated Hospital of Harbin Medical University, Harbin, China; 2 Department of Colorectal Surgery, Cancer Institute & Hospital, Chinese Academy of Medical Sciences, Peking Union Medical College, Beijing, China; Taipei Medical University, TAIWAN

## Abstract

**Background:**

With the expanding population of colorectal cancer (CRC) survivors in the United States, one concerning issue is the risk of developing second primary malignancies (SPMs) for these CRC survivors. The present study attempts to identify the incidence characteristics of SPMs after diagnosis of first primary colon cancer (CC) and rectal cancer (RC).

**Methods:**

189,890 CC and 83,802 RC cases were identified from Surveillance, Epidemiology and End Results Program (SEER) database. We performed rate analysis on incidence trend of SPMs in both CC and RC. Expected incidence rates were stratified by age, race and stage, calendar year of first CRC diagnosis and latency period since first CRC diagnosis. The standardized incidence ratios (SIRs), measure for estimating risk of SPMs, were calculated for CC and RC respectively.

**Results:**

The trends of incidence of SPMs in both CC and RC were decreasing from 1992 to 2012. Both CC and RC survivors had higher risk of developing SPMs (SIRCC = 1.13; SIRRC = 1.05). For CC patients, the highest risks of SPM were cancers of small intestine (SIR = 4.03), colon (SIR = 1.87) and rectum (SIR = 1.80). For RC patients, the highest risks of SPMs were cancers of rectum (SIR = 2.88), small intestine (SIR = 2.16) and thyroid (SIR = 1.46). According to stratified analyses, we also identified incidence characteristics which were contributed to higher risk of developing SPMs, including the age between 20 and 40, American Indian/Alaska Native, localized stage, diagnosed at calendar year from 2002 to 2012 and the latency between 12 and 59 months.

**Conclusions:**

Both CC and RC survivors remain at higher risk of developing SPMs. The identification of incidence characteristics of SPMs is extremely essential for continuous cancer surveillance among CRC survivors.

## Introduction

Colorectal cancer (CRC) incidence rate is increasing and mortality is declining in a large number of countries worldwide over recent decades, these trends are most likely attributed to popularized cancer screening and wider availability of state of the art therapy approaches [1–4]. Owing to the expanding population of CRC survivors, one concerning issue is their long-term survival status, especially the risk of developing second primary malignancies (SPMs) [5, 6]. Accumulated evidence suggested an elevated risk in patients with CRC for development of a variety of SPMs [7–9]. Intensive treatments for initial CRC, normal aging, and continuous exposed to carcinogens are considered to be major risk factors for the increased incidence of SPMs [10–12].

From a clinical perspective, assessing risk factors of SPMs could facilitate strategies for prevention and early detection of SPMs. More importantly, it is very significant to identify which subgroups of CRC patients are more susceptible to develop SPMs in considering different epidemiological and clinical features of first primary CRC. It was well established that the biological characteristics and prognosis were different between colon cancer (CC) and rectal cancer (RC) [13]. However, most of current studies evaluated risk of SPMs by considering CC and RC as the same condition, which might generate some misunderstandings of results [14].

In this study, we used data from Surveillance Epidemiology and End Results (SEER) cancer registries [15], and respectively evaluated the risk of SPMs after diagnosis of CC and RC in the United States between 1992 and 2012. This was done to determine whether the risk of SPMs is different between CC and RC, and to assess which epidemiological and clinical features of first primary CRC are more likely to lead to occurrence of SPMs.

## Methods

### Date source

The SEER database was utilized to access the processed publically available data since January 1, 1992 to December 31, 2012 from 13 registries (New Mexico, San Francisco-Oakland, Atlanta, San Jose-Monterey, Rural Georgia, Connecticut, Detroit, Hawaii, Iowa, Utah, Los Angeles, Seattle-Puget Sound and the Alaska Native Tumor Registry). The demographic and incidence data collected by the SEER registries approximately cover 28 percent of the US population, which are considered to be the representative of the US population as a whole. To ensure that SPMs can be separated from metastases and recurrences of first primary CRC, the SEER strictly adheres to the coding rules of topography or histology classification of International Classification of Diseases for Oncology third edition (ICD-O-3). The subsite of CC includes cecum, ascending colon, hepatic flexure, transverse colon, splenic flexure, descending colon, and sigmoid colon, the subsite of RC includes rectosigmoid junction and rectum. Appendix and large intestine without specific location information have been excluded from this study.

### Study population

The first primary CRC included patients with only one primary CRC as well as the first CRC of patients with multiple primary cancers. We collected invasive CRC patients who were diagnosed at the age of more than 20 years to ensure that young patients could be captured in this cohort for their increasing proportion in the population of cancer patients. We excluded first primary CRC cases including patients: 1) diagnosed with unknown age, 2) reported only on death or autopsy certificate only, 3) being stage of in situ. In addition, the SPMs diagnosed during six months period after the primary diagnosis were also excluded to minimize misclassification of undetected synchronous cancers and metastases.

### Ethics statement

The study design was approved by the Ethics Committee of Second Affiliated Hospital of Harbin Medical University. The National Cancer Institute’s SEER Program Database is an openly accessed database. Cancer diagnoses are reportable diseases to the cancer registries, including those that provide data to SEER. Therefore, the authors can obtain cancer cases and population data from website for SEER. Patients’ records were anonymized and de-identified prior to analysis.

### Statistical analyses

Incidence rates of SPMs were calculated and expressed per 100,000 person-years. The incidence trends were expressed by estimating the annual percent change (APC). The APC is described by joinpoint regression which is one statistical method for evaluating the trends of increase or decrease over continuous periods of time [16]

Multiple primary standardised incidence ratios (MP-SIRs) were applied as one measure to estimate risk of SPMs. SIR was defined here as the ratio of the observed incidence of SPMs among CRC to the expected incidence in US general population of the SEER ascertainment area. A determination of statistical significance of SIRs was based on p-value<0.05 (two-sided). 95% confidence intervals were calculated by Poisson exact methods for the ratio of observed events to expected events. The total number of expected events is defined as:

$$E^{*}\;=\;\overset{M}{\underset{k=1}{\sum }}E_{K}^{*}\;=\;\overset{M}{\underset{k=1}{\sum }}t_{k}\lambda_{k}^{*}$$

The person-time from the study group is allocated among M cells defined by the cross-classification of various adjustment variables such as gender, race and attained age group. *t*_*k*_ represents the person-time, and *λ*_*k*_ represents the standard rate for the kth cell, where k = 1, 2… M.

Expected incidence rates were stratified by age at first primary CRC diagnosis (20–39, 40–59, ≥60), race (white, black, American Indian/Alaska Native, Asian or Pacific Islander), calendar year of first CRC diagnosis (1992–2001, 2002–2012), latency period since first CRC diagnosis (6–11 months, 12–59 months, 60–119 months, ≥120 months), stage (localized, regional, distant). SIRs were calculated by CC and RC separately. All analyses were conducted using SEER*Stat software version 8.2.1 (available at: http://seer.cancer.gov/seerstat/; latest release April 8, 2015).

## Results

### Study population characteristics

In this study, we identified 240,584 eligible individuals who were diagnosed with first primary CRC between 1992 and 2012 in the 13 SEER registries. Among them, 164,748 were CC and 75,836 were RC. Of these 240,584 primary CRC patients, 20,064 CC patients and 7,667 RC patients subsequently developed SPMs (Table 1). Among these SPMs, the male patients occupied 57.7% in CC and 61.3% in RC. 66.7% of CC patients and 67.4% of RC patients were diagnosed between 1992 and 2001. The proportions in some characteristics, such as age over 60 years, white, and localized stage, were obviously greater in the CRC patients who developed SPMs. Most of SPMs were diagnosed within the period from 12 to 59 months after first primary CRC diagnosis. At the latency of 60–119 months and ≥120 months from first primary CRC diagnosis, the incidence of SPMs in both CC and RC were gradually decreased.

**Table 1:** Variables of CC and RC patients who developed SPMs.

Variable	Patients who developed SPMs			
	Colon (n = 20,064)		Rectum (n = 7,667)	
**Sex**				
**Male**	11,568	57.7%	4,698	61.3%
**Female**	8,496	42.3%	2,969	38.7%
**Age at first CRC diagnosis**				
**20–39**	208	1.0%	125	1.6%
**40–59**	3,452	17.2%	1,888	24.6%
**≥60**	16,404	81.8%	5,654	73.8%
**Race**				
**White**	16,211	80.8%	6,153	80.3%
**Black**	2,125	10.6%	730	9.5%
**American Indian/ Alaska Native**	85	0.4%	25	0.3%
**Asian or Pacific Islander**	1,643	8.2%	759	9.9%
**Calendar year**				
**1992–2001**	13,391	66.7%	5,169	67.4%
**2002–2012**	6,673	33.3%	2,498	32.6%
**Stage at first CRC diagnosis**				
**Localized**	6,015	30.0%	2,647	34.5%
**Regional**	5,001	24.9%	1,430	18.7%
**Distant**	554	2.8%	183	2.4%
**Unstaged/unknown**	8,494	42.3%	3,407	44.4%
**Latency (months)**				
**6–11**	1,598	8.0%	514	6.7%
**12–59**	9,463	47.2%	3,411	44.5%
**60–119**	5,764	28.7%	2,352	30.7%
**≥120**	3,239	16.1%	1,390	18.1%

### Incidence trend for SPMs

**Fig 1:**
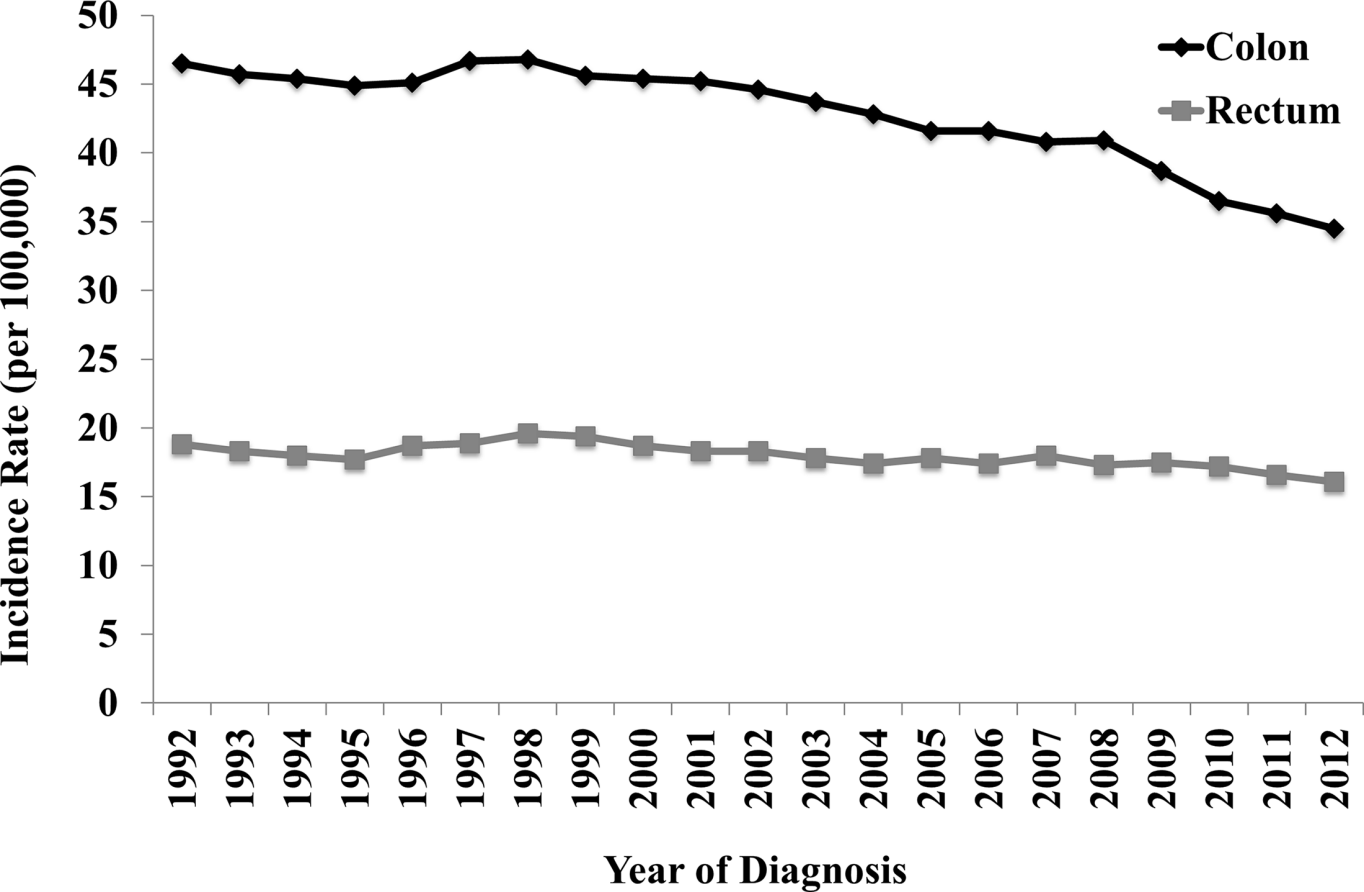
The incidence rates of SPMs from 1992 to 2012.

The incidence rates of SPMs in both CC and RC were significantly decreasing over the period of 1992–2012. For CC patient, the incidence rate declined from 46.5 per 100,000 to 34.5 per 100,000. For RC patients, the incidence rate also decreased from 18.8 per 100,000 to 16.1 per 100,000 (Fig 1). In addition, our results also showed that the APCs of incidence trend for SPMs were annually declined by 1.3% in CC and 0.6% in RC.

### SIRs for SPMs

Compared with cancer incidence in the general population, individuals with CC and RC had higher risks overall for SPMs (SIRCC = 1.13, SIRRC = 1.05) (Table 2). For CC patients, the most predominantly elevated SIR was cancer of small intestine (SIR = 4.03), followed by colon (SIR = 1.87) and rectum (SIR = 1.80). In addition, there were also significantly elevated incidences of SPMs in stomach, lung and bronchus, corpus uteri, prostate, kidney and thyroid. However, the risks of SPM in liver, gallbladder, ovary, brain and lymphatic and hematopoietic diseases were significantly decreased. For RC patients, the most markedly elevated SIR was the cancer of rectum (SIR = 2.88), followed by small intestine (SIR = 2.16) and thyroid (SIR = 1.46). Furthermore, the risks of SPMs increased in colon, lung and bronchus, corpus uteri, urinary bladder, and decreased only in prostate.

**Table 2:** Risk of SPMs in colon and rectal cancer patients.

SPMs	Colon		Rectum	
	Obs	SIR (95% CI)	Obs	SIR (95% CI)
**All sites**	20,064	1.13 (1.12–1.15)*	7,667	1.05 (1.02–1.07)*
**Esophagus**	226	1.14 (1.00–1.30)	86	1.01 (0.81–1.25)
**Stomach**	462	1.21 (1.10–1.32)*	161	1.05 (0.89–1.23)
**Small intestine**	311	4.03 (3.59–4.50)*	68	2.16 (1.68–2.74)*
**Colon**	2,824	1.87 (1.80–1.94)*	792	1.38 (1.29–1.48)*
**Rectosigmoid junction and rectum**	914	1.80 (1.69–1.92)*	610	2.88 (2.66–3.12)*
**Liver**	203	0.86 (0.74–0.98)*	94	0.86 (0.70–1.05)
**Gallbladder**	40	0.69 (0.50–0.94)*	17	0.81 (0.47–1.30)
**Pancreas**	612	1.07 (0.98–1.15)	202	0.92 (0.80–1.06)
**Lung and bronchus**	2,868	1.07 (1.03–1.11)*	1,200	1.11 (1.05–1.17)*
**Female breast**	1,839	0.99 (0.94–1.03)	647	0.93 (0.86–1.00)
**Cervix uteri**	68	1.13 (0.88–1.43)	27	1.12 (0.74–1.63)
**Corpus uteri**	506	1.39 (1.28–1.52)*	170	1.21 (1.03–1.41)*
**Ovary**	161	0.75 (0.64–0.87)*	68	0.88 (0.68–1.11)
**Prostate**	3,204	1.04 (1.00–1.07)*	1,039	0.71 (0.66–0.75)*
**Kidney**	506	1.16 (1.06–1.26)*	194	1.01 (0.87–1.16)
**Urinary bladder**	1,109	1.03 (0.97–1.09)	510	1.16 (1.06–1.27)*
**Brain**	128	0.82 (0.68–0.97)*	61	0.91 (0.69–1.16)
**Thyroid**	205	1.38 (1.20–1.59)*	103	1.46 (1.19–1.77)*
**Bones and joints**	12	0.82 (0.43–1.44)	9	1.44 (0.66–2.73)
**Soft tissue including heart**	104	1.12 (0.92–1.36)	50	1.31 (0.98–1.73)
**All lymphatic and hematopoietic diseases**	1,489	0.93 (0.88–0.98)*	596	0.93 (0.86–1.01)
**Others**	2,273	0.95 (0.91–0.99)*	963	0.99 (0.93–1.05)

Obs: Observed events; SIR: Standard incidence ratio; CI: Confidence interval;

*P<0.05.

#### Age at first CRC diagnosis

**Fig 2:**
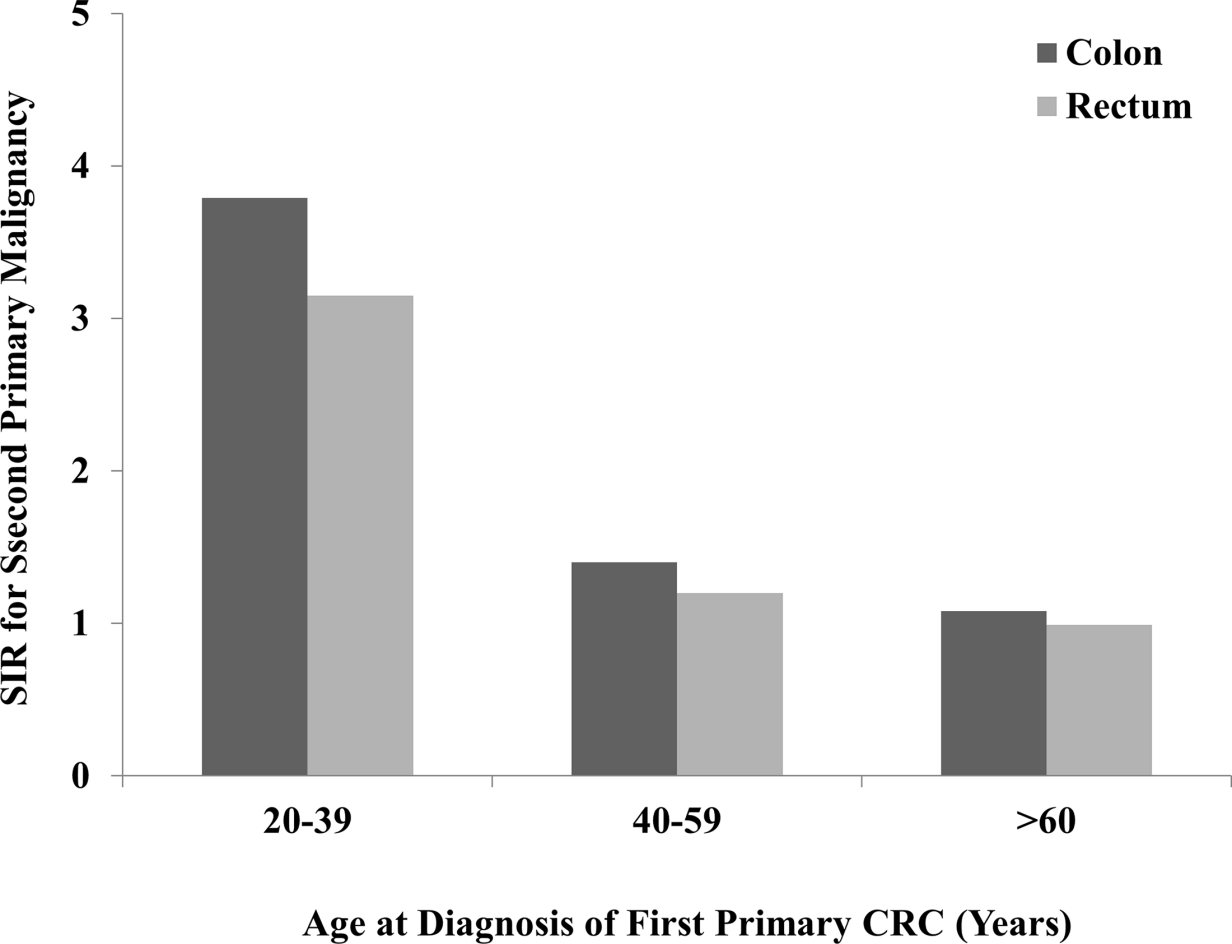
SIRs of SPMs in colon and rectal cancer patients by age.

Age is usually considered as one crucial prognostic factor in CRC, so we compared the SIRs in three different age subgroups including 20–39 years, 40–59 years and ≥60 years, to determine whether age could influences the risk of SPMs. The results showed that both CC and RC patients aged 20–39 years had predominantly higher risks of developing SPMs in all site in contrast to other elder age groups (Fig 2). The similar trends of incidence were also observed in SPMs of small intestine, colon, rectum, pancreas, lung and bronchus, and corpus uteri. In addition, CC patients who aged 20 to 39 had significantly increased risk of developing SPMs of small intestine, colon, rectum and corpus uteri, with SIRs>10.0. The similar trend was also observed in the SPM of rectum in RC patients (Table 3). The detailed information including observed events and the CIs of SIRs were listed in S1 Text.

**Table 3:** SIRs of SPMs in colon and rectal cancer patients, by age.

SPMs	Colon			Rectum		
	20–39	40–59	>60	20–39	40–59	>60
**All sites**	3.79*	1.40*	1.08*	3.15*	1.20*	0.99
**Esophagus**	3.15	1.03	1.15	0	0.84	1.06
**Stomach**	3.66	1.81*	1.13*	6.66*	1.14	1.01
**Small intestine**	42.45*	6.89*	3.37*	5.46	3.99*	1.59*
**Colon**	16.71*	3.55*	1.67*	9.63*	1.86*	1.27*
**Rectosigmoid junction and rectum**	18.54*	3.15*	1.51*	19.18*	4.28*	2.35*
**Liver**	0	1.00	0.82*	3.52	0.99	0.79
**Gallbladder**	0	0.80	0.69*	0	0.98	0.79
**Pancreas**	9.20*	1.23	1.03	7.60*	0.97	0.89
**Lung and bronchus**	3.09*	1.19*	1.05*	4.56*	1.37*	1.05
**Female breast**	1.36	1.00	0.98	1.33	0.85*	0.95
**Cervix uteri**	1.97	1.28	1.05	0	0.91	1.34
**Corpus uteri**	10.33*	2.22*	1.11	5.94*	1.19	1.15
**Ovary**	2.70	1.30	0.64*	2.42	1.05	0.80
**Prostate**	1.25	1.05	1.03	0.98	0.71*	0.70*
**Kidney**	1.62	1.33*	1.11*	2.26	1.12	0.96
**Urinary bladder**	4.05*	1.24*	1.00	1.48	1.44*	1.12*
**Brain**	3.86*	0.97	0.76*	2.60	0.94	0.87
**Thyroid**	1.91	1.74*	1.20	1.40	1.74*	1.26
**Bones and joints**	0	1.15	0.76	0	3.44*	0.68
**Soft tissue including heart**	6.82*	1.79*	0.97	4.56	1.66	1.16
**All lymphatic and hematopoietic diseases**	1.51	1.02	0.92*	2.85*	0.97	0.91*
**Others**	2.82*	1.29*	0.89*	2.27*	1.28*	0.90*

*P<0.05

#### Race

**Fig 3:**
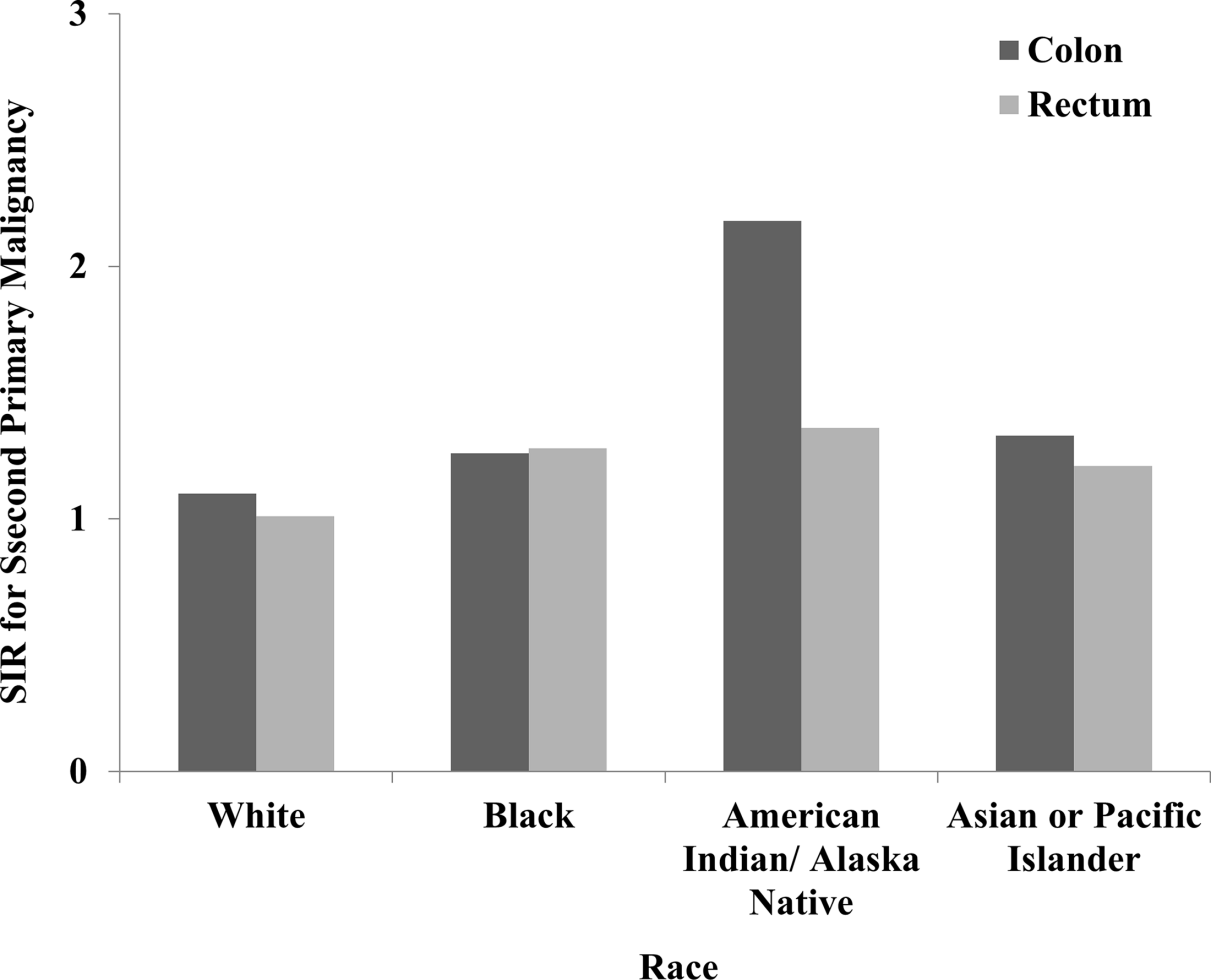
SIRs of SPMs in colon and rectal cancer patients by race.

There were significant changes in SIRs between the different races for SPMs in all sites (Fig 3). The highest risk for SPMs belongs to American Indian/Alaska Native in CC and black in RC. In CC patients, the risk of developing SPMs of small intestine, colon, rectum, liver, gallbladder, corpus uteri, ovary, prostate, kidney, and brain all were higher in black in contrast with white. However, the risk was significantly reduced in black patients compared with white for malignancies of lung and bronchus, and thyroid. As for RC patients, the higher risk of SPMs including small intestine, colon, rectum, lung and bronchus and urinary bladder were observed in black patients (Table 4).

**Table 4:** SIRs of SPMs in colon and rectal cancer patients, by race.

SPMs	Colon				Rectum			
	White	Black	American Indian/Alaska Native	Asian or Pacific Islander	White	Black	American Indian/Alaska Native	Asian or Pacific Islander
**All sites**	1.10*	1.26*	2.18*	1.33*	1.01	1.28*	1.36	1.21*
**Esophagus**	1.11	1.36	2.72	1.09	1.09	0.30	0	0.96
**Stomach**	1.12	1.30	1.60	1.47*	1.02	1.10	0	1.16
**Small intestine**	3.90*	4.44*	7.16	4.51*	1.89*	4.03*	0	2.23
**Colon**	1.82*	2.09*	4.06*	1.95*	1.38*	1.61*	1.94	1.19
**Rectosigmoid junction and rectum**	1.69*	2.75*	2.44	1.84*	2.59*	5.44*	2.53	3.38*
**Liver**	0.83*	0.74	1.19	0.98	0.90	0.89	0	0.78
**Gallbladder**	0.70*	0.95	0	0.46	0.73	1.90	0	0.68
**Pancreas**	1.05	0.98	0	1.32*	0.85*	1.26	0	1.23
**Lung and bronchus**	1.07*	1.06	1.40	1.12	1.07*	1.31*	1.87	1.29*
**Female breast**	0.97	1.06	0.95	1.14	0.92	0.98	1.11	0.94
**Cervix uteri**	1.20	1.02	0	0.95	1.22	0.89	0	0.88
**Corpus uteri**	1.32*	1.36*	8.63*	2.32*	1.21*	0.70	2.79	1.72*
**Ovary**	0.72*	0.69	4.50	1.10	0.83	1.37	0	1.04
**Prostate**	0.99	1.22*	2.17*	1.19*	0.65*	0.92	1.75	0.94
**Kidney**	1.09	1.63*	1.10	1.24	1.00	0.94	2.20	1.17
**Urinary bladder**	1.00	1.00	3.40*	1.39*	1.17*	1.58*	0	0.81
**Brain**	0.74*	1.18	3.84	1.65	0.90	0.70	0	1.23
**Thyroid**	1.34*	1.15	3.12	1.79*	1.36*	2.03	0	1.89*
**Bones and joints**	0.65	0	0	4.32*	1.12	2.47	0	4.22
**Soft tissue including heart**	1.07	1.27	9.08*	1.30	1.28	0.83	9.67	1.71
**All lymphatic and hematopoietic diseases**	0.92*	0.91	2.32	1.10	0.90*	1.24	1.44	1.05
**Others**	0.93*	0.97	1.80	1.22*	0.95	1.33*	1.13	1.29*

*P<0.05

#### Stage of first CRC diagnosis

**Fig 4:**
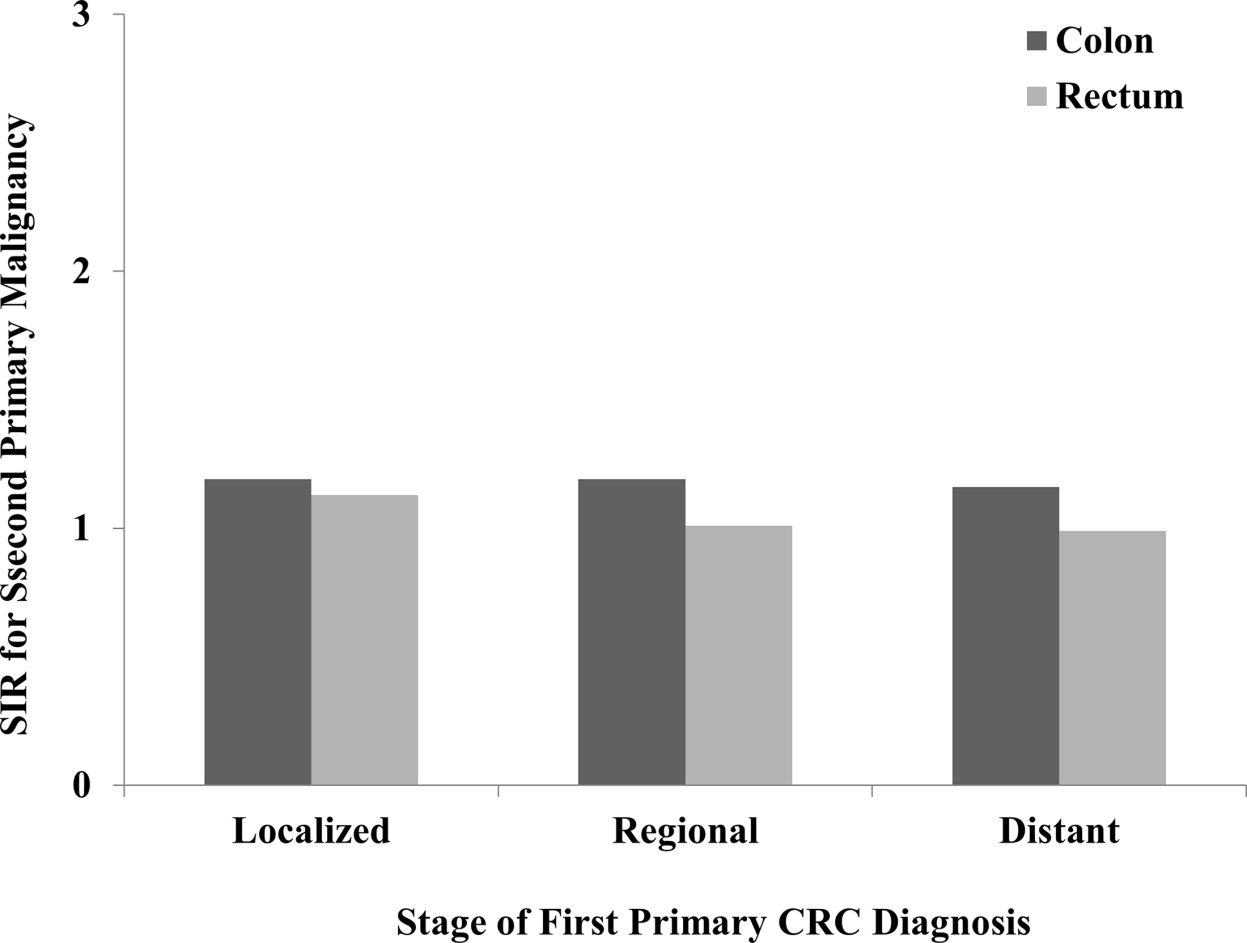
SIRs of SPMs in colon and rectal cancer patients by stage.

Whether incidence of SPMs increases with the progression of first primary CRC, we divided first primary CC and RC into three subgroups which contained localized stage, regional stage and distant stage. The differences of SIRs among these three groups were compared in CC and RC separately. As compared with localized and regional stage, the first primary CC and RC diagnosed in distant stage did not reasonably increase risks of developing SPMs in all sites (Fig 4). However, incidences of SPMs in small intestine, colon and rectum did increase corresponding to the progression of first primary CC, and only colon as SPM exhibited the similar trend in RC patients. Taking into account other SPMs, there was no significant change based on different stages (Table 5).

**Table 5:** SIRs of SPMs in colon and rectal cancer patients, by stage.

SPMs	Colon			Rectum		
	Localized	Regional	Distant	Localized	Regional	Distant
**All sites**	1.19*	1.19*	1.16*	1.13*	1.01	0.99
**Esophagus**	1.36*	1.07	0.38	0.81	1.68*	0.47
**Stomach**	1.42*	1.11	0.96	1.12	0.84	1.11
**Small intestine**	3.06*	6.22*	10.01*	1.50	3.42*	0
**Colon**	1.87*	2.31*	3.13*	1.37*	1.58*	1.95*
**Rectosigmoid junction and rectum**	1.59*	2.51*	4.64*	4.60*	2.92*	3.58*
**Liver**	1.06	0.83	0.93	0.98	1.10	0.30
**Gallbladder**	0.92	0.72	0.73	0.60	1.53	0
**Pancreas**	1.07	1.04	1.19	1.02	0.91	0.76
**Lung and bronchus**	1.18*	1.13*	0.84	1.12*	1.05	0.94
**Female breast**	1.05	0.99	0.68*	0.94	0.87	0.79
**Cervix uteri**	1.13	1.08	1.01	0.65	1.29	2.79
**Corpus uteri**	1.47*	1.40*	2.30*	1.09	1.66*	0.51
**Ovary**	0.78	0.67*	0.86	0.81	0.54	2.00
**Prostate**	1.13*	1.03	0.71*	0.85*	0.46*	0.55*
**Kidney**	1.29*	1.02	1.05	1.00	1.09	0.92
**Urinary bladder**	1.05	0.98	1.21	1.12	1.10	0.92
**Brain**	1.05	0.76	0	1.00	0.81	1.10
**Thyroid**	1.74*	1.51*	1.85	1.49*	2.30*	0.80
**Bones and joints**	0.70	1.67	0	0.96	0.78	5.61
**Soft tissue including heart**	1.00	1.12	1.19	1.80*	1.42	0
**All lymphatic and hematopoietic diseases**	0.95	0.90*	0.46*	1.00	0.76*	0.76
**Others**	1.02	0.92	0.90	1.06	0.99	1.22

*P<0.05

#### Calendar year of first CRC diagnosis

**Fig 5:**
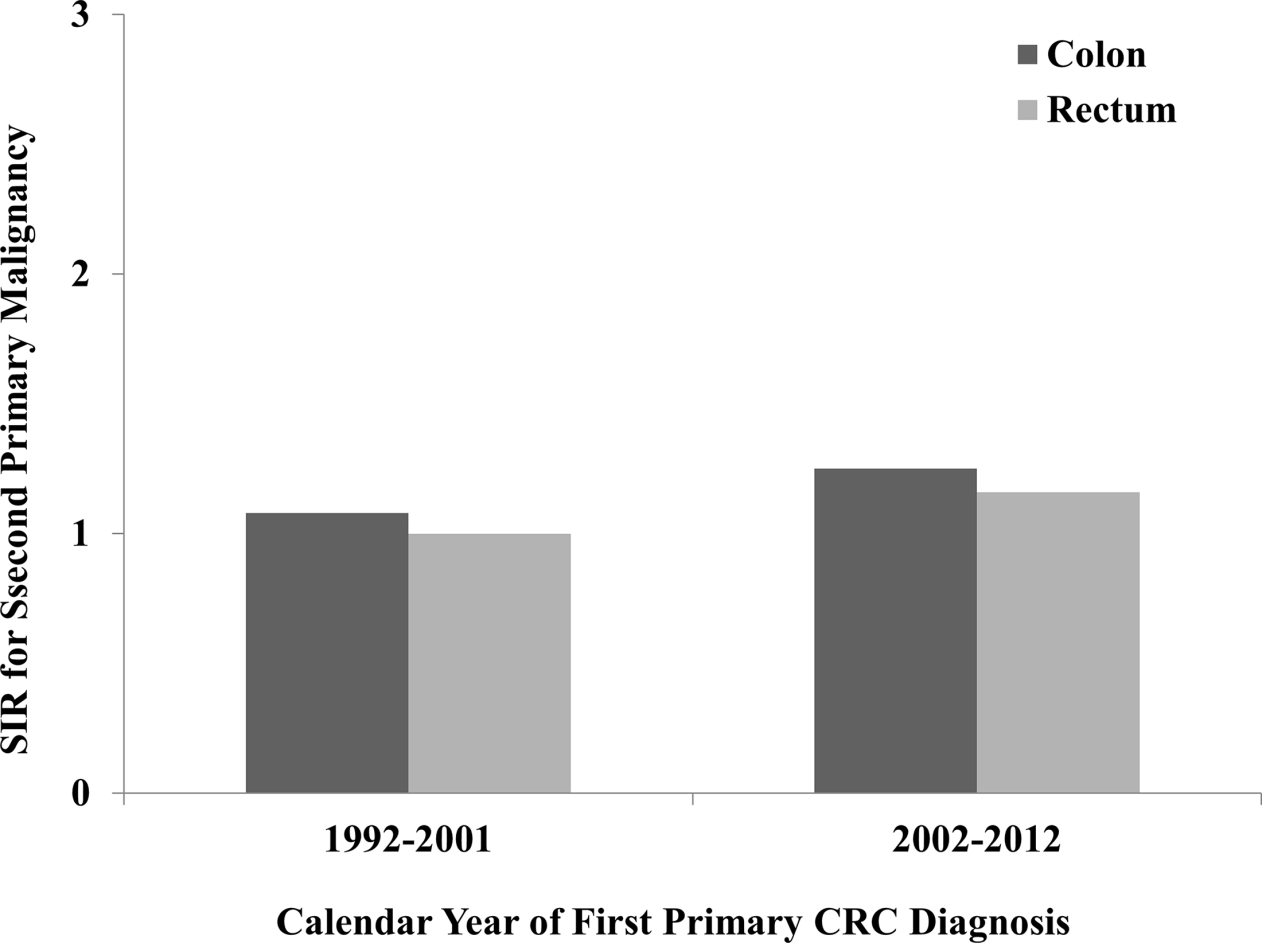
SIRs of SPMs in colon and rectal cancer patients by calendar year.

The data collected by the SEER registries covers a long period of time, during which diagnostic strategies and treatment methods may be altered substantially. Therefore, we evaluated whether there were differences by two calendar periods of the data (1992–2001 vs 2002–2012). There was significantly increased risk of developing a SPM in CC. For RC patients, the increased risk was only observed during the period between 2002 and 2012 (Fig 5). The dramatically elevated incidences of SPMs which included small intestine, colon, rectum, lung and bronchus, kidney and thyroid were observed in both CC and RC during the period of 2002 to 2012. On the contrary, the risks of developing lymphatic and hematopoietic diseases decreased recently (Table 6).

**Table 6:** SIRs of SPMs in colon and rectal cancer patients, by calendar year.

SPMs	Colon		Rectum	
	1992–2001	2002–2012	1992–2001	2002–2012
**All sites**	1.08*	1.25*	1.00	1.16*
**Esophagus**	1.09	1.25	0.99	1.08
**Stomach**	1.20*	1.22*	1.02	1.13
**Small intestine**	3.51*	5.04*	1.99*	2.52*
**Colon**	1.64*	2.48*	1.34*	1.52*
**Rectosigmoid junction and rectum**	1.45*	2.73*	1.68*	6.06*
**Liver**	0.80*	0.95	0.70*	1.14
**Gallbladder**	0.60*	0.92	0.67	1.17
**Pancreas**	1.05	1.10	0.86	1.06
**Lung and bronchus**	1.03	1.17*	1.10*	1.12*
**Female breast**	0.96	1.04	0.94	0.91
**Cervix uteri**	1.05	1.33	1.26	0.81
**Corpus uteri**	1.34*	1.50*	1.21*	1.20
**Ovary**	0.74*	0.76	0.97	0.66
**Prostate**	1.02	1.08*	0.70*	0.71*
**Kidney**	1.13*	1.21*	0.86	1.30*
**Urinary bladder**	1.01	1.06	1.22*	1.01
**Brain**	0.79*	0.87	0.82	1.11
**Thyroid**	1.00	1.93*	1.16	1.90*
**Bones and joints**	0.71	1.05	1.42	1.47
**Soft tissue including heart**	1.20	0.95	1.00	1.97*
**All lymphatic and hematopoietic diseases**	0.95	0.88*	0.96	0.86*
**Others**	0.93*	1.01	0.96	1.05

*P<0.05

#### Latency since first CRC diagnosis

**Fig 6:**
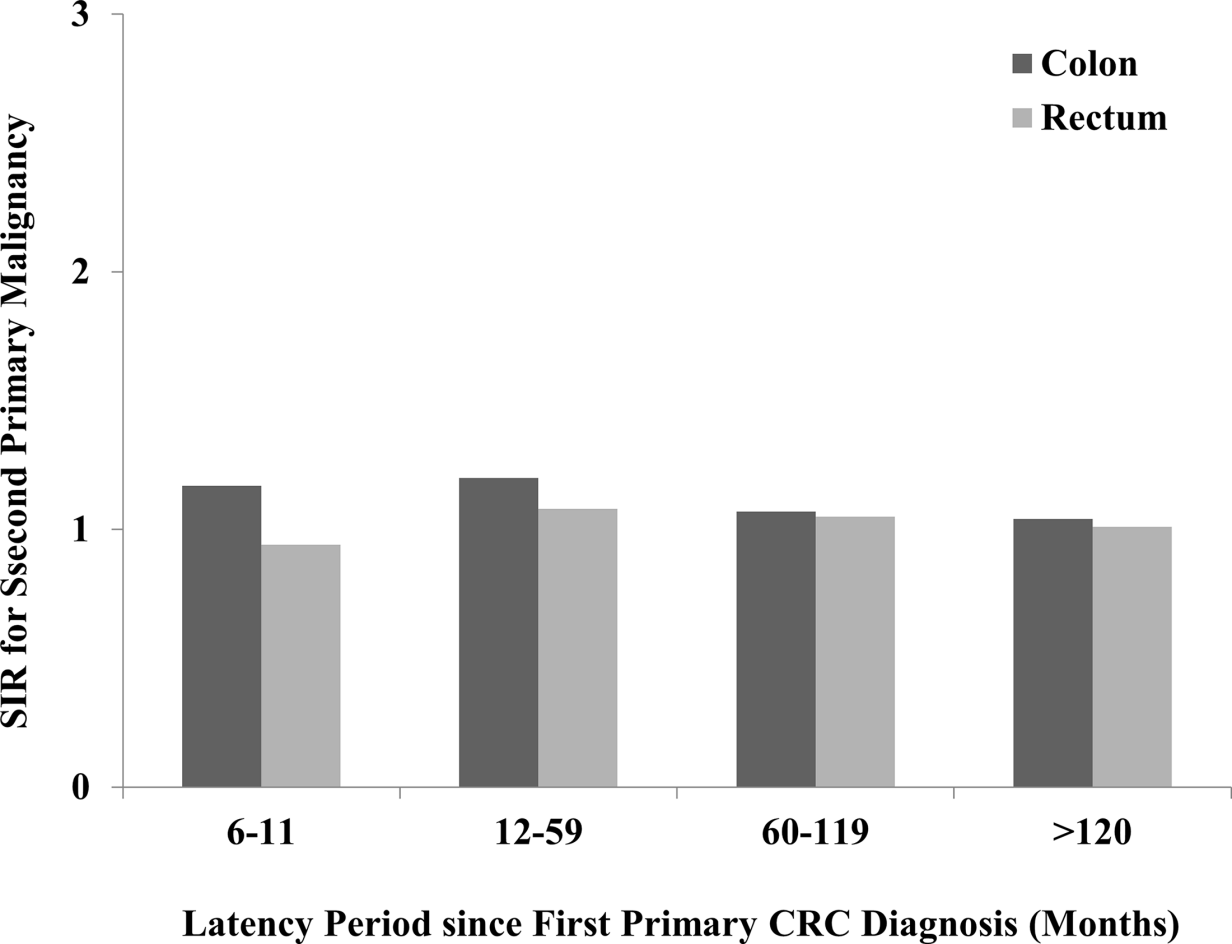
SIRs of SPMs in colon and rectal cancer patients by latency.

The highest risk of developing a SPM was the latency between 12–59 months from the first primary CRC diagnosis (Fig 6). With the extension of latent period after 60th month, the risk of SPMs gradually decreased in both CC and RC. After 11 months of first CC diagnosis, there were dramatically increased risks for cancers of small intestine, colon, rectum, cervix uteri, corpus uteri and thyroid. After the latency of 120 months, the risk was reduced for most of these malignancies among CC and RC patients. However, cancers of small intestine, colon, rectum, corpus uteri and urinary bladder still had relatively increased risks of SPMs after 120 months of latency (Table 7).

**Table 7:** SIRs of SPMs in colon and rectal cancer patients, by latency.

SPMs	Colon				Rectum			
	6–11	12–59	60–119	>120	6–11	12–59	60–119	>120
**All sites**	1.17*	1.20*	1.07*	1.04*	0.94	1.08*	1.05*	1.01
**Esophagus**	0.94	1.22	1.07	1.15	1.46	0.94	1.10	0.86
**Stomach**	1.23	1.27*	1.14	1.15	0.87	1.00	1.20	0.99
**Small intestine**	6.17*	5.30*	2.26*	3.25*	1.85	2.94*	1.43	1.83
**Colon**	2.57*	2.26*	1.36*	1.40*	1.59*	1.56*	1.21*	1.16
**Rectosigmoid junction and rectum**	2.22*	2.40*	1.15	1.07	2.13*	4.24*	1.83*	1.49*
**Liver**	0.70	0.86	0.92	0.81	1.04	0.87	0.68	1.05
**Gallbladder**	0.24	0.68	0.90	0.55	0	1.27	0.31	0.92
**Pancreas**	0.93	1.06	1.06	1.13	0.86	0.78*	1.06	1.01
**Lung and bronchus**	0.98	1.12*	1.07*	0.99	0.80	1.08	1.24*	1.08
**Female breast**	0.88	0.97	1.04	0.99	0.91	0.94	0.91	0.93
**Cervix uteri**	2.01*	1.24	0.64	1.17	1.87	1.05	1.30	0.56
**Corpus uteri**	1.54*	1.54*	1.21*	1.25	0.64	0.87	1.43*	1.93*
**Ovary**	0.87	0.85	0.61*	0.64*	0.17*	0.74	0.85	1.56
**Prostate**	0.90	1.07*	1.04	1.01	0.72*	0.66*	0.74*	0.77*
**Kidney**	1.69*	1.17*	0.98	1.20	1.47	1.23	0.70*	0.87
**Urinary bladder**	0.94	1.01	1.12*	0.95	0.87	1.13	1.22*	1.23*
**Brain**	1.08	0.68*	0.91	0.90	0.20	1.03	0.83	1.03
**Thyroid**	2.69*	1.44*	1.24	0.95	2.14*	1.65*	1.25	1.12
**Bones and joints**	0.90	1.09	0.45	0.76	0	1.11	1.58	2.53
**Soft tissue including heart**	1.22	1.17	0.94	1.27	1.51	1.15	1.86*	0.76
**All lymphatic and hematopoietic diseases**	0.83	0.88*	1.00	0.97	0.93	0.86*	1.05	0.90
**Others**	0.91	0.94	0.98	0.95	0.73*	1.01	1.05	0.95

*P<0.05

## Discussion

In this study, we observed that overall incidence trends of SPMs in both CC and RC were significantly decreased from 1992 to 2012. The potential reason for this interesting finding is mainly due to the enhanced cancer screening, which is beneficial for the early detection and treatment of precancerous lesions. However, both CC and RC survivors had higher risks of developing SPMs compared with the general US population. The rise in overall cancer risk was consistent with previous studies [5, 8, 17]. For SPMs of CC, increased risks were observed for stomach, small intestine, colon, rectum, lung/bronchus, kidney, prostate, corpus uteri, and thyroid malignancies. Of these, the SIR was the most pronounced with respect to risk of small intestine, this result was similar with the previous study which was regardless of the anatomic location of the first primary CRC [5]. In contrast, CC survivors had lower risk of developing liver, gallbladder, brain, ovary malignancies and lymphatic and hematopoietic diseases. For RC survivors, they had higher risk of developing small intestine, colon, rectum, lung/bronchus, corpus uteri, urinary bladder, and thyroid malignancies. The decreased risk was only observed for prostate malignancy. The gradually increased observed events of certain SPMs may be also due in part to enhanced surveillance and screening after CRC diagnosis, which resulted in the detection of latent malignancies [18].

Previous studies have shown that risks of SPMs after CRC differed by location of first tumor [9, 19]. In this work, our results also indicated that incidences of SPMs were different between CC and RC. The potential reasons for this pattern are uncertain, but discrepancies in clinicopathological features, biological characteristics and treatment strategies between CC and RC may contribute to differences of SPMs. Proximal CC always showed larger tumor size, higher T-stage, higher tumor grade and more frequent mucinous histologic subtype compared to RC [20]. Proximal CC showed more frequent methylation of Type 2 markers, CIMP+, MSI, BRAF mutations and lower frequencies of LOH and global hypomethylation, MGMT methylation was more frequent in RC [21]. Specifically, differences of treatment strategies between CC and RC could be considered as crucial impact factors for incidences of SPMs. For example, neoadjuvant radiotherapy has become a standardized treatment for locally advanced RC, but not for CC, which could be the potential reason for the increased risk of SPM in urinary bladder, because of its exposure to radiation beams. Furthermore, these evidences also suggested that cancer surveillance after CRC might need to be individualized based on the anastomotic sites of the first CRC.

In this study, we interestingly found that the risk for liver cancer decreased in patients with CC, and the mechanism of the declined risk of liver cancer is still unclear. However, recent study has revealed that intestinal microbiota and Toll-like receptors (TLRs) could induce the inflammatory and fibrogenic responses, and TLR4 activation by lipopolysaccharide from the gut microbiota could provoke the injury- and inflammation-driven tumor promotion, which further contribute to hepatocarcinogenesis [22]. Therefore, we speculated that resection of colon and rectum may reduce chance of developing liver cancer by interfering gut microbiota.

Age, one of the most crucial prognostic factors for incidence of CRC, also has close relationship to SPMs. In this study, we found that 76.6% CC patients and 82.6% RC patients were aged more than 60 years, the risk of CRC in the elderly were much greater than the young. This result was consistent with previous studies [23, 24]. However, taking into account of the risk of developing SPMs after CRC in different age subgroups, the trends of SPMs has obviously decreased with age increased. The SIRs were 3.79 in CC and 3.15 in RC in subgroup of age between 20 and 39, which exhibited obviously increased risk compared with the elder subgroups. In CC patients, the risks in some SPMs, including small intestine, colon, rectum and corpus uteri, have increased to even more than 10 times comparing with the general population. Although young CRC patients are more likely to have mucinous or poorly differentiated cancers [25], whether they carry poor prognosis remains controversial [26]. In considering possible reasons contributing to the observed increased risk of young CRC patients, genetic susceptibility in cases with familial cancer syndromes, including Lynch syndrome (previously known as hereditary non-polyposis colorectal cancer, HNPCC) and familial adenomatous polyposis which characteristically have onset at younger age than sporadic malignancy. Furthermore, patients with Lynch syndrome also had significantly increased risk of developing malignancies of stomach, small intestine, corpus uteri, ovary and pancreas [27–29]. Familial adenomatous polyposis is also presented with a very high risk of developing CRC, relative high risk of small intestinal and thyroid cancer [30, 31].

Among CRC survivors, American Indian and Alaska Native patients tended to have the greatest risk of developing SPMs, followed by black and Asian or Pacific Islander., especially for CC patients. White patients had the lowest risk of developing SPMs in both CC and RC. The influences of racial disparities in CRC incidence and survival have been studied for several decades [32, 33], and the inequality in access to medical care played a major role in the racial disparities seen in treatment of CC in general population [34]. However, there were few studies comparing the risk of developing SPMs among different race. In white patients, the risk of developing SPMs was relatively low in most of cancers. This might reflect differences in cancer screening, early treatment of precancerous lesions, genetic predisposition, environmental exposures and these combined factors between different races. Future investigation should be required to explore specific reasons for these differences.

It is well known that prognosis of CRC in distant stage is obviously worse than localized and regional stage. However, it is still unclear that whether the risk of developing SPMs is similar in different staged CRC patients. In this study, we compared the SIRs in different CRC stage. Compared with regional and distant stages, SIRs in localized stage were obviously higher in both CC and RC, which was contrast to our expectations. The potential reason is that patients in localized stage always experience much longer lifetime, which make them face more chances of developing SPMs during their lifetime. In addition, the SPMs including small intestine, colon and rectum had an opposite trend, showing that SIRs in distant stage were higher than localized and regional stage. Perhaps the advanced CRC had worse biological characteristics which made them more easily be suffered from second intestinal cancer again.

The risk of developing SPMs obviously decreased with latency prolonged in both CC and RC. For RC patients, the risk of developing SPMs in latency of more than 60 months is similar with the general population. Most recurrences and metastases always occur within 60 months after the diagnosis of CRC. The probabilities of recurrence and metastasis of CRC after 60 months are relatively low. Therefore, postoperative follow-up within 60 months is a very crucial period for CRC. That may be the potential reason that most SPMs were diagnosed in the latency within 60 months. However, according to our results, many CRC survivors are still presented with high risk of developing SPMs after 60 months of follow up, such as cancers of small intestine, colon, rectum, corpus uteri and urinary bladder.

Potential limitations in this study include lack of detailed information, such as the treatment strategies for CRC, the lifestyle factors and comorbidities of CRC patients. In this study, misclassification of the treatment received probably would have been non-differential and further have led to an underestimate of the actual risk of SPMs associated with radiation therapy. In addition, the number of statistical tests performed to estimate the risk of SPMs by tumor type does increase the likelihood of observing a significant finding by chance alone, suggesting that these results should be interpreted with some caution. Finally, some results in this study are different from other studies [19, 35]. One reason account for these variations may be due to the objective differences with regard to area, ethnic, environment, and genetic characteristics, as well as the subjective evaluation factors including coding rule, reporting system and follow up scheme among cancer registers throughout the world.

## Conclusion

In conclusion, the overall risk of developing SPMs between CC and RC is almost consistent, but there still has some discrepancies. The results in this study indicate that, the risk of developing SPMs among both CC and RC patients is higher than that in the general population, and CRC survivors remain at risk of developing certain malignancies, particularly for malignancies originated from the same endoderm. The subgroup of young CRC patients has much higher risk of developing SPMs compared with the elder CRC patients. Significant racial disparities are also found, indicating that white CRC patients are less likely to experience SPMs than other race counterparts. The localized CRC patients are most likely to develop SPMs than regional and distant stage counterparts. SPMs are most likely to occur during the latency period from 12 to 59 months after the initial diagnosis of CRC. All of these findings have significantly clinical implications for effective prevention and continuous surveillance of SPMs among CRC survivors.

## Supporting Information

S1 Text The detailed information including observed events and the CIs of SIRs. (XLS)
